# Abundance of active ingredients in sea-buckthorn oil

**DOI:** 10.1186/s12944-017-0469-7

**Published:** 2017-05-19

**Authors:** Aleksandra Zielińska, Izabela Nowak

**Affiliations:** Faculty of Chemistry, Adam Mickiewicz University in Poznań, Umultowska 89b, 61-614, Poznań, Poland

**Keywords:** Vegetable oils, Fatty acids, Sea buckthorn oil, Gamma-linolenic acid, Human health, Aging process

## Abstract

Vegetable oils are obtained by mechanical extraction or cold pressing of various parts of plants, most often: seeds, fruits, and drupels. Chemically, these oils are compounds of the ester-linked glycerol and higher fatty acids with long aliphatic chain hydrocarbons (min. C14:0). Vegetable oils have a variety of properties, depending on their percentage of saturation. This article describes sea-buckthorn oil, which is extracted from the well characterized fruit and seeds of sea buckthorn. The plant has a large number of active ingredients the properties of which are successfully used in the cosmetic industry and in medicine. Valuable substances contained in sea-buckthorn oil play an important role in the proper functioning of the human body and give skin a beautiful and healthy appearance. A balanced composition of fatty acids give the number of vitamins or their range in this oil and explains its frequent use in cosmetic products for the care of dry, flaky or rapidly aging skin. Moreover, its unique unsaturated fatty acids, such as palmitooleic acid (omega-7) and gamma-linolenic acid (omega-6), give sea-buckthorn oil skin regeneration and repair properties. Sea-buckthorn oil also improves blood circulation, facilitates oxygenation of the skin, removes excess toxins from the body and easily penetrates through the epidermis. Because inside the skin the gamma-linolenic acid is converted to prostaglandins, sea-buckthorn oil protects against infections, prevents allergies, eliminates inflammation and inhibits the aging process. With close to 200 properties, sea-buckthorn oil is a valuable addition to health and beauty products.

## Background

Vegetable oils, as a rich source of fatty acids, have gained a common recognition and found applications in the market of medical and cosmetic products [1–6]. Fatty acids contained in these oils create an occlusive film on the skin which reduces transepidermal water loss (TEWL), thus contributing to maintaining the correct hydration of epidermis [3, 7]. Moreover, fatty acids protect, regenerate and soften stratum corneum, relieve inflammation and ensure an appropriate structure of the skin intercellular cement [3, 6]. Depending on the percentage content of individual ingredients, particularly fatty acids, the effect of oils on skin and human health may vary [1–4]. For example, the deficiency of oil results in skin being deprived of the sufficient protective layer and causes flaking [6]. Vegetable oils, while playing the part of a base in cosmetic products, protect against excessive water loss through skin mainly by forming an occlusive film which covers the epidermis [3, 4, 6]. In inflammations, oils lower turgor of skin and reduce the perception of pain [3, 6]. Triglycerides of long-chain fatty acids play a significant part in appropriate functioning of the human body [1, 2, 4, 7]. Vegetable oils play a significant part in biological synthesis of cell membrane components or icosanoids (eicosanoids: prostaglandins, prostacyclins, thromboxanes, leucotrienes) [3]. Oils take part in transport and oxidation of cholesterol [7]. Fatty acid deficiency weakens blood vessels, lowers immunity, disturbs the process of blood clotting and favours the development of atherosclerosis [7–9]. One of the natural glycerides is sea-buckthorn oil which has a rich chemical composition and unique properties [9–14]. This oil is obtained as a result of mechanical cold pressing or extraction from fruit or seeds of the plant [12]. The latest scientific studies confirm the presence of many active ingredients in the extract of common sea-buckthorn (*Hippophaes rhamnoides*) obtained by cold extraction from the fruit of the plant [10, 11, 14], including antioxidants, vitamin C, flavonoids, polyphenols and polysaccharides. Nowadays, both the fruit of sea-buckthorn (*Fructus Hippophae*) and its seeds (*Semen Hippophae*) are not only raw materials for food industry, a medicinal product, but also commonly used ingredient of cosmetic products, the properties of which are beneficial for the skin [12]. After taxonomic, chemical and sensory tests of common sea-buckthorn fruit carried out at a university in Finland, where sea-buckthorn is considered to be a plant with special pro health properties, it was proved that the fruit of *Hippophaes rhamnoides* significantly increases the level of beneficial high-density lipoprotein (HDL) cholesterol fraction [11]. These results may help to prevent cardiovascular diseases in healthy people [9]. Interestingly, sea-buckthorn fruit was known and valued already in the ancient times, in particular, in traditional Asian medicine. It should be noted that the generic name of the plant, *Hippophae*, originated in ancient Greece, where sea-buckthorn was fed to horses to make their coats nicer and more shiny (Greek *hippos* – horse; *phaos* – shiny) [14, 15].

## Botanical description of the product

Common sea-buckthorn (*Hippophaes rhamnoides*), also called a Siberian pineapple, is a thorny, dioecious shrub (or tree) in the oleaster family (*Elaeagnaceae*) growing up to 7 m high [12, 16, 17]. It has a smooth or sometimes cracked bark. The name sea-buckthorn may be hyphenated to avoid confusion with the buckthorns in *Rhamnaceae* family. Sea-buckthorn is also known as sandthorn, sallowthorn or seaberry [18]. The plant grows in Europe, Caucasus, Asia Minor and Central Asia, Siberia, China and Tibet [16, 19, 20]. Sea-buckthorn is the most common species in the *Hippophae* family: *H. goniocarpa* *,* *H. gyantsensis* *,* *H. litangensis* *,* *H. neurocarpa* *,* *H. rhamnoides* *L.,* *H. salicifolia* *,* *H. tibetana* *, H. sinensis*, which grow from the Atlantic coast of Europe to northwestern Mongolia and northwestern China [20, 21]. In western Europe sea-buckthorn is confined to sandy sea cliffs, dunes and mountain slopes. In central Asia it is found in dry and sandy areas, often as a subalpine shrub. In Poland it is found usually on the Baltic coast, where it tolerates salty soils and forms dense thickets [16–20]. The shrub is tolerant of both drought and frost as well as air pollution [12]. Common sea-buckthorn flowers in late April and early May. The plant has long lanceolate leaves covered in silvery hairs underneath. The shrub produces a large number of small, green and brown flowers which grow together in racemes. After the flowering period, they turn into tasty and nutritious round berries, usually yellow or orange, which ripen in September. Inside the fruit there is a smooth, small stone which has a long groove and covers an oily seed [12, 17–22]. Sea-buckthorn fruits are bitter and sour in taste and have a delicate aroma, resembling that of a pineapple [12, 14, 15]. The berries are a rich source of vitamins C, E and P as well as malic acid and citric acid. Harvesting sea-buckthorn fruit is very difficult due to dense thorn arrangement among the berries. Therefore, sometimes the only way to get valuable fruit is to remove the entire branch of the shrub, which reduces future crops [16, 17, 20]. For this reason berries can only be harvested once every two years [17, 23]. Sea-buckthorn berries have an impressive vitamin content [12, 24, 25]. They contain mainly vitamin C [11–14, 20] (approximately 900 mg%, depending on the variety), but also vitamin A, that is alpha- and beta-carotene (up to 60 mg%) and a mixture of other carotenoids (up to 180 mg% in total). Moreover, the berries contain tocopherols, that is vitamin E (110 to 160 mg%), folic acid (up to 0.79 mg%) and vitamin B complex group, i.e. B_1_ (0.035 mg%), B_2_ (up to 0.056 mg%) and B_6_ [14, 15, 24–28]. The fruits contain flavonoids (with an effect of vitamin P), catechins and procyanidins, cyclitols, phospholipids, tannins, sugars: galactose, fructose, xylose, approx. 3.9% organic acids (maleic acid, oxalic acid, malic acid, tartaric acid) [11–14, 20], phenolic acids, e.g. ferulic acid as well as fatty oil (the content of oil in common sea-buckthorn berry pulp is up to 8 wt.% and in seeds up to 12.5 wt.%) [24–29]. The content of vitamin C depends on the variety of the plant and its geographical location. For example, sea-buckthorn growing in Europe in coastal dunes contains 120–315 mg% of vitamin C in fresh fruit, and the species growing in the Alps contains much more vitamin – 405-1100 mg%. Chinese sea-buckthorn fruits (*Hippophae sinensis*) are richest in vitamin C, with ascorbic acid content of up to 2500 mg% [14–18, 21, 27–29]. Moreover, the content of carotenoids with an effect of vitamin A is also high. The content of beta-carotene is 40–100 mg% and other carotenoids such as lycopene, cryptoxanthin, physalien, zeaxanthin account for 180–250 mg% [14, 15, 30–32]. When the berries are pressed, the resulting juice separates into three layers. The upper layer is a thick orange cream, the middle layer contains a mixture of saturated and unsaturated fatty acids, and the lower layer is a juice which is a source of fat used for cosmetic purposes [32–35]. Two upper layers can be processed and used in making of skin care creams, and the bottom layer is usually used in food industry as syrup. Currently, the highly nutritious ingredients of common sea-buckthorn berries are tested for their application in medicine, i.e. in treatment of inflammations, cancers and as adjunctive treatment after chemotherapy [33–39]. Bark and leaves of sea-buckthorn used to be applied to treat diarrhea and dermatological conditions, whereas berry oil applied topically or taken orally softened the skin [40]. In Indian, Chinese and Tibetan medicines sea-buckthorn fruits were added to medicines, as their ingredients were thought to have a beneficial effect on the function of the alimentary, respiratory and circulatory systems. Nowadays, many studies confirm the practices of Asian doctors from hundreds of years ago [41–44]. Physical and chemical properties of sea-buckthorn seed oil are contained in Table 1.

**Table 1:** Physical and chemical properties of sea-buckthorn seed oil [61]

The parameter	Value
color, absorptivity (L/g·cm)	
232 nm	2.89 ± 0.03
270 nm	0.64 ± 0.02
303 nm	0.41 ± 0.02
410 nm	0.06 ± 0.02
diene value	3.16 ± 0.01
triene value	0.070 ± 0.002
*p*-anisidine value	34.19 ± 0.06
peroxide value (mequiv/kg)	20.68 ± 0.06
saponification number	190.00 ± 1.63
viscosity (mpas·s)	44.0 ± 0.5
carotenoid content (mg/100 g)	41.1 ± 13.4
tocopherol content (mg/100 g)	
α	155.0 ± 7.0
β	16.4 ± 1.7
γ	134.9 ± 2.8
δ	11.3 ± 1.4
vitamin E equiv. (mg/100 g)	175.0 ± 8.0

## Obtaining the sea-buckthorn seed oil

**Fig. 1:**
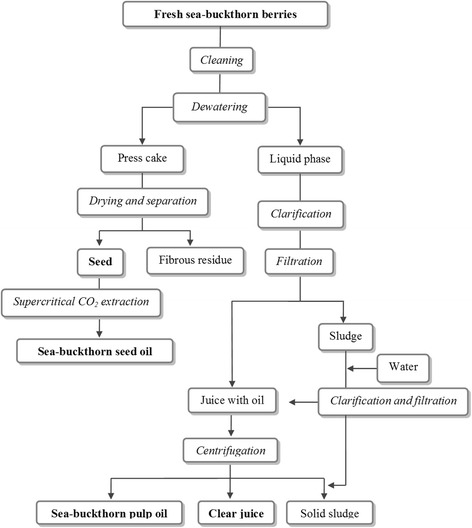
A schematic diagram shows of the patented method for processing of sea buckthorn berries for seed oil, pulp oil and juice

Common sea-buckthorn oil can be obtained from two parts of the plant [45, 46]. Figure 1 presents one of the exemplary and patented method processing of fresh sea buckthorn berries for seed oil, pulp oil and juice [47]. Firstly, sea-buckthorn oil may be extracted in the process of mechanical cold pressing of seeds which contain up to 12.5 wt.% of oil [12–15, 48–52]. Secondly, the oil is obtained by extraction or in cold pressing of fruit pulp which contains 8–12 wt.% oil. The obtained fractions are filtered [12–15, 46, 48, 53, 54]. The two types of oils differ significantly in terms of appearance and properties. For example, of all vegetable oils sea-buckthorn fruit oil has the highest content of palmitooleic acid (omega-7) of 30 to 35 wt.%, which is not as high in sea-buckthorn seed oil [53–55]. The oil obtained from juicy berries is a thick dark orange or red-orange liquid with a characteristic smell and taste (sourish, if pressed from fruit pulp) [53, 55, 56]. Sea-buckthorn seed oil and fruit oil differ significantly in terms of their content of active ingredients [45–55]. However, both oils contain a wide range of essential unsaturated fatty acids (UFA), in particular unique palmitooleic acid (C16:1) which is highly valued in cosmetology. Both oils abound in tocopherols, tocotrienols and plant sterols [50–52, 55, 56]. Unlike seed oil, pulp sea-buckthorn oil has a high content of carotenoids [56]. In Mongolia, Russia and China pulp oil is used topically in treatment of skin burns [58–60]. The oil has been introduced to the local markets by cosmetic companies in anti-aging cosmetics and oral care preparations.

## Composition of chemical compounds

Sea-buckthorn fruit oil is characterised by a unique content of fatty acids compared to other vegetable oils [61–63]. In particular, it should be noted that this oil contains rare palmitooleic acid (omega-7) which is a component of skin lipids and stimulates regenerative processes in the epidermis and wound healing. Thanks to it, sea-buckthorn oil activates physiological skin functions and reduces scars [64–66]. Used orally it supports treatment of gastric, duodenal and intestine ulcers, while applied topically its soothes and reduces skin burns (caused by sun exposure or radiotherapy), chafed skin, bedsores and trophic skin changes [64–66]. Additionally, sea-buckthorn oil contains saturated fatty acids in the form of palmitic acid C16:0 (30–33 wt.%) and stearic acid C18:0 (<1 wt.%), and it has a wide range of essential unsaturated fatty acids (UFA), in particular so called PUFA (polyunsaturated fatty acids) [12, 61–63]. They include alpha-linolenic acid (omega-3) C18:3 (30 wt.%), gamma-linolenic acid (omega-6) C18:3 (35.5 wt.%), linolic acid (omega-6) C18:2 (5–7 wt.%), oleic acid (omega-9) C18:1 (14–18 wt.%) and eicosanoic acid (omega-9) C20:1 (2 wt.%) [3, 12, 14, 15, 61–63] (Table 2). Such a high content of unique gamma-linolenic acid (GLA) has a significant effect on the transport of nutrients. GLA is also a very important ingredient for skin, because as a building material for components of intercellular cement it binds epidermis cells. It is also a component of phospholipids which build cell membranes [14, 15]. Gamma-linolenic acid improves blood circulation which positively affects the supply of nourishment and oxygen to skin, and it removes excess toxins which as a result improves skin structure, appearance and tone. GLA contained in sea-buckthorn oil easily penetrates to deeper skin layers where it is converted to prostaglandins. Therefore, GLA effectively protects skin against infections, counteracts allergies, relieves inflammations and slows down the ageing process [67, 68]. Moreover, skin deprived of this rare omega-6 acid becomes drier, less elastic and susceptible to any lesions [68]. The presence of linolic acid (omega-6), which is a component of intercellular cement, results in stimulation of cellular regeneration and regulates the functions of skin sebaceous glands [69]. The composition of fatty acids with various properties ensures multidirectional effects of sea-buckthorn oil in different layers of epidermis. On the other hand, a high content of saturated fatty acids (above 30 wt.%) causes the oil to soften the epidermis and protect and secure it against transepidermal water loss [61, 63, 67–69].

**Table 2:** Composition of fatty acids in sea-buckthorn oil [3, 12, 14, 15, 61, 63]

Common name	Systematic name	Content in wt.%	General formula	Numerical symbol	Omega family
Saturated fatty acids					
Palmitic acid	Hexadecanoic acid	30–33	CH_3_(CH_2_)_14_COOH	C16:0	-
Stearic acid	Octadecanoic acid	<1	CH_3_(CH_2_)_16_COOH	C18:0	-
Unsaturated fatty acids					
Palmitoleic acid	(Z)-9-hexadecenoic acid	30–35	C_16_H_30_O_2_	16:1	7
Oleic acid	(Z)-9-octadecenoic acid	14–18	C_18_H_34_O_2_	18:1	9
Linoleic acid (LA)	(Z,Z)-9,12-octadecadienoic acid	5–7	C_18_H_32_O_2_	18:2	6
α-Linolenic acid (ALA)	(Z,Z,Z)-9,12,15- octadecatrienoic acid	30	C_18_H_30_O_2_	18:3	3
γ-linolenic acid (GLA)	(Z,Z,Z)-6,9,12- octadecatrienoic acid	35	C_18_H_30_O_2_	18:3	6
Gondoic acid	(Z)-11-eicosenoic acid	2	C_20_H_38_O_2_	20:1	9

### Saturated fatty acids

The most common saturated fatty acids in vegetable oils include palmitic, stearic, myristic and arachidic acids. They ensure high stability of the oil and its resistance to oxidation [3]. Sea-buckthorn oil contains palmitic and stearic acids [3, 12]. These acids form a protective occlusion on the skin which strengthens the effect of a protective barrier. They provide appropriate turgor and firmness of skin, and have smoothing and softening properties [3, 12, 14, 15, 61, 63].

### Unsaturated fatty acids

This group of acids includes fatty acids in the form of colourless liquids, with double bonds. For most of them all double bonds are in a *cis* configuration [3]. Nowadays, two main classes of unsaturated fatty acids are distinguished. They are monounsaturated FA (omega-9; ω-9; n-9 acids) and polyunsaturated FA (omega-6; ω-6; n-6 acids). Polyunsaturated FA have at least two double bonds and 18 carbon atoms in an alkyl chain [3]. Sea-buckthorn oil contains linolic acid (LA) and alpha-linolenic acid (ALA) from this group which cannot be produced by a human body due to a lack of certain enzymes. Other polyunsaturated acids found in the oil, i.e. gamma-linolenic acid, oleic acid and palmitoleic acids, can be produced by the body providing there is no enzymatic defect in the course of metabolic changes [3, 12]. Linolic acid is considered to be the most important of all omega-6 acids as other acids in this group, i.e. ALA or GLA can be obtained from it [3, 7, 70–72].

### Complex lipids

Sea-buckthorn oil also contains the complex lipids which include:

- phospholipids and glycolipids that exhibit skin moisturizing and soften the epidermis, improve elasticity of the skin, reduce inflammation of the skin, accelerate skin regeneration and cell renewal. For example lecithin (also known as phosphatidylcholine), belonging to the group of phospholipids, has skin renewing and moisturizing properties, as well as it slows the aging process and furthermore, it removes excessive oil (sebum) from the hair. According to the Shugam et al., the total phospholipids content in sea-buckthorn oil was 1 wt.%. The lecithin content in this oil was detected by thin layer chromatography [73]. Other scientific research [74] also have confirmed that the oil from sea buckthorn pericarp contains from 0.2–0.5 to 1 wt.% of phospholipids. Among them, 5.8 wt.% has been estimated to lecithin.
- sterols, which strengthens the lipid barrier of the skin, protects from harmful substances of external origin and reduces the excessive water loss through the epidermis, thereby improving the skin elasticity and firmness. The petroleum-ether technique was used to extract the highest amount of β-sitosterol (576.9 mg/100 g oil), being the major sterol compound throughout the berry and constitutes 57–83 wt.% of total sterols [75]. In turn, β-sitosterol including with campesterol and stigmasterol were present in the pulp oil with the latter having together the highest contribution (97 wt.%). Using the petroleum-ether technique, the quantity of cholesterol (4.5 mg/100 g oil) was also extracted [76]. In the sea-buckthorn oil has proven the minor amount (less than 1 wt.%) of liposomes, allowing the introduction of active substances into the skin or ceramides that provide the proper hydration and smooth the skin, as well as they provide skin firming and regeneration [77].

### Other bioactive compounds and their significance for a human health

In addition, sea-buckthorn oil contains many active substances, through which this oil has many different properties (Table 3). In particular vitamins A, C, E, F, P and B complex are present in the oil [12, 14, 15, 34, 45]. Vitamin A, found in the form of carotenoids (approx. 200 mg/100 g), provides regenerative and anti-wrinkle properties of the oil [31–34, 57]. Vitamin C, the content of which is 15 times higher than in orange fruit (approx. 695 mg/100 g), has an antioxidative effect [58–60] and protects against harmful UVA and UVB radiation [12, 14, 15, 38, 56, 78]. It also evens out the skin tone. The presence of vitamin E in the form of tocopherols (approx. 200–600 mg/100 g) and minerals and flavonoids strengthens the walls of capillary blood vessels. Sea-buckthorn oil also contains sterols, fruit acids (malic acid, citric acid), phenolic compounds, tannins, phospholipids, anthocyanins, sugars, pectins and mineral salts including sulfur, selenium, copper and zinc [12, 14, 15, 50–52]. The importance for human health of sea-buckthorn oil have been proved by in vivo tests and have shown in Table 4.

**Table 3:** Composition of other bioactive ingredients contained in sea-buckthorn oil and their significance for a human health

Name of ingredient	Quantity	Significance
polyphenols	120–550 mg%	antioxidant properties
phenolic acids: - salicylic - p-coumaric - m-coumaric - p-hydroxyphenyl lactic acid - gallic acid	71 wt.% of polyphenols	participation in the creation of dyes and protection against the development of undesirable microflora [83]
flavonoids		inhibition of thrombosis and hypertension [84–86], and promotion of wound healing [87]
- flavan-3-ols - (catechin, epicatechin, gallocatechin, epigallocatechin) - kaempferol - quercetin - isorhamnetin - myricetin - rutin - proanthocyanidins		antioxidants, stabilization of ascorbic acid [88]
sterols	1 wt.%	reduction of blood cholesterol level, importance in the treatment of burns, huge contribution in the synthesis of steroid hormones and other biologically active compounds [89, 90]
phytosterols		
sitosterol	48–53 wt.% of phytosterols	
tocopherols (vitamin E)	110 mg%	antioxidants, according to the study, the degree of fruit ripeness effects on the content of tocopherols [91, 92]
α-tocopherol	62–68 wt.% of tocopherols	
δ-tokoferol	32–37 wt.% of tocopherols	
macronutrients		they are energy-providing chemical substances consumed by organisms in large quantities [93]
potassium	168–219 mg%	affects muscle spasms
magnesium	8.3–9.5 mg%	with calcium is responsible for the proper functioning of the nervous system
calcium	5–7.2 mg%	for the proper functioning of the muscular system
micronutrients		they are required by organisms throughout life in small quantities to orchestrate a range of physiological functions [93]
iron	1.24 mg%	component of hemoglobin, myoglobin and coenzymes many enzymes involved, among others, in the formation of ATP
zinc	0.25 mg%	participates in various stages of protein biosynthesis, ingredient of insulin (also plays an important role in the storage of the pancreas), regulates the concentration of vitamin A is used in the formation of bone, stimulates growth and tissue repair (wound healing)
manganese		necessary for proper development of tissue (especially bone) and for the functioning of the central nervous system
copper	0.006 mg%	cofactor of many enzymes
nickel	0.015 mg%	component of urease - an enzyme decomposing urea into ammonia and carbon dioxide
vitamins		they have diverse biochemical functions [93]
vitamin C	900 mg%	antioxidant, participates in the synthesis of collagen fibers, removes free radicals and strengthens immunity.
vitamin A	60 mg%	antioxidant
vitamin E (tocopherols)	up to 160 mg%	antioxidant
vitamin B1	0.016–0.035 mg%	function as enzyme cofactors (coenzymes) or the precursors for them
vitamin B2	0.03–0.05 mg%	
vitamin B6 (Folic acid)	up to 0.079 mg%	
vitamin K1	0.9–15 mg%	normalizes blood clotting, and is essential for preventing osteoporosis and normal renal function
vitamin D		prevents rickets and osteomalacia
carotenoids	7.94–28.16 mg%	antioxidants and plant pigments, anticancer properties [91, 94–98]
δ-carotene	14–25 wt.% of carotenoids	
γ-carotene	30 wt.% of carotenoids	
lycopene	30 wt.% of carotenoids	
zeaxanthin and other carotenoids	15 wt.% of carotenoids

**Table 4:** Sea-buckthorn oil and its importance for human health proved by in vivo tests –literature review

Function of oil	Reference
✓ has antiatherogenic properties ✓ protects the heart ✓ has antiaggregative properties ✓ can be used in the treatment of peptic ulcer disease ✓ has antioxidative properties ✓ antiinflammatory ✓ protects cardiovascular disease ✓ has commercial applications due to the high level of ω-7	[84]
✓ in the treatment of burns, chilblains, bedsores, difficult healing of wounds	[99]
✓ it is proved its application in the treatment of peptic ulcer disease.	[100]
✓ exhibits an anti-atherosclerotic effect.	[101]
✓ protects cardiovascular disease and inhibits the risk factors.	[102]
✓ has antioxidant, anti-ulcerogenic and hepato-protective actions, and its berry oil is reported to suppress platelet aggregation.	[103]
✓ has the antihypertensive effect due to the flavones extracted from seed residues of *Hippophae rhamnoides L*.	[104]
✓ has dermal wound healing activity	[105]
✓ reduces the increase of the osmotic concentration in tear film during the cold season and positively affects the dry eye symptoms.	[106]
✓ has significant hepatoprotective effects ✓ can be used as a food supplement against liver diseases	[107]

## Significance of fatty acids found in sea-buckthorn oil for skin

Linolic acid found in sea-buckthorn oil plays a significant role in skin. It strengthens the lipid barrier of the epidermis in dry skin and protects against transepidermal water loss. Additionally, LA regulates skin metabolism [3, 14, 15, 68–70]. Linolic acid is also a natural component of sebum. In patients with acne prone skin a decrease in the content of linolic acid in sebum was noted. As a result blackheads and spots form. Linolic acid used in the care of oily and problematic skin can stimulate the function of sebaceous glands, unblock pores and limit the number of blackheads. LA is also used for the production of intercellular cement [3, 69, 72]. Gamma-linolenic acid, which is also found in sea-buckthorn oil, is formed as a result of action of delta-6-desaturase enzyme in a process of metabolic changes of linolic acid. Together with alpha-linolenic acid, GLA is a component of cell membranes or mitochondrial membranes of human cells [3, 7, 68, 72]. GLA and ALA are also responsible for normal intra- and intercellular transport (including the transfer of stimuli in the neuronal network forming the brain) [3, 7, 70, 79]. It is assumed that unsaturated fatty acids, in particular in omega-3 group (mainly EPA and DHA), inhibit the development of neoplastic tumours as well as growth of neoplastic tissue and its later metastasis [80]. It was also proved that these acids can reduce post-inflammatory substances, induced by a harmful UV radiation. These compounds reduce the effects of sunburns, accelerate regenerative processes of the damaged lipid barrier of the epidermis and soothe irritation [64–66, 68, 78]. Omega fatty acids: omega-9 (oleic acid), omega-6 (linolic acid), and omega-3 (alpha-linolenic acids) lower transepidermal water loss and improve the skin hydration level [69, 71, 72]. Unsaturated FA play a part of receptors which stimulate the synthesis of barrier lipids of skin and proteins – precursors of a natural hydrating factor [70–72].

## Sea-buckthorn oil in cosmetic products

Sea-buckthorn oil is used in cosmetic industry as an ingredient of preparations for mature skin [12, 70]. It is most commonly found in anti-ageing and anti-wrinkle products, as it is a great antioxidant [13–15, 24, 36, 39, 58, 70]; it also firms and tones sagging skin smoothing out wrinkles [70]. Sea-buckthorn oil is also appropriate in care of dry, irritated (e.g. after sunbathing), rough, flaking and itchy skin [38, 68]. It is used as auxiliary product in treatment of frostbites and skin damage [54, 66] resulting from exposure to UV radiation, x-rays and chemical compounds [4, 38, 78]. Sea-buckthorn oil stimulates wound healing (including necrotic wounds), stimulating regeneration and processes of forming new healthy epidermis, and moreover collagen synthesis [81]. This oil reduces bedsores, treats eczema and reduces spots, acne, allergic and inflammatory lesions of the skin [40, 58, 66]. The oil is used as a soothing agent after cosmetic procedures e.g. peelings, baths, masks, hair removal. Its presence in shampoos, hair conditioners or preparations used after dying or permanent wave treatment guarantees recovery, supports regeneration of damaged hair, restores its elasticity and ensures smoothness. Due to a high content of unsaturated fatty acids [61, 68] and related fast rancidity process of sea-buckthorn oil is recommended that it is used in the form of capsules for cosmetic products [62, 81]. It is also significant that sea-buckthorn oil, thanks to its intensive colour, improves skin tone after direct application on skin, giving it a fresh and healthy appearance [31, 34, 57].

## Sea-buckthorn oil for human health

Sea-buckthorn oil as well as extracts from its fruit are used as an adjunctive therapy in treatment of many diseases [1, 2, 4, 8–12, 27, 37, 42, 43]. Sea-buckthorn oil has a soothing effect in inflammation of the alimentary system, duodenum or in diarrhea [37, 38]. It is successfully used in treatment of chronic gastric ulcer disease and also in inflammations of vagina and cervix and in cervical erosion [1, 2, 37]. This oil relieves symptoms of rheumatoid disease, lowers cholesterol level, stops small bleeding and lowers the risk of thrombophlebitis [8, 9, 11, 37]. Sea-buckthorn oil is also recommended in febrile diseases, in particular caused by viruses and bacteria [37]. It is safe to use by pregnant and breastfeeding women [14, 15, 78]. The oil is effective in treatment of dermatoses and any skin diseases and it supports the process of granulation of wounds that are difficult to heal [64–66]. Sea-buckthorn oil strengthens the structure of hair therefore it is used as an effective remedy against hair loss or even balding. As a natural source of well absorbed vitamin C, this oil is used as an adjunctive treatment in a number of conditions which require an increased amount of ascorbic acid and as an agent supporting the function of the immune system [14, 15, 67, 82]. Thanks to a high content of carotenoids and tocopherols [12, 82] sea-buckthorn oil can be used in treatment of burns, frostbites, bedsores and skin damage, e.g. resulting from the exposure to sun or x-rays [4, 38, 78].

## Summary

**Sea-buckthorn oil contains approximately 190 bioactive substances including:** *saturated fatty acids*- palmitic acid C16:0, stearic acid C18:0, *unsaturated fatty acids*- eicosanoic acid ω-9 C20:1, oleic acid ω-9 C18:1, palmitoleic ω-7 C16:1, linolic acid ω-6 C18:2, alpha-linolenic acid ω-3 C18:3, gamma-linolenic acid ω-6 C18:3, sterols, approx. 14 vitamins: A, C, D, E, F, K, P, and B complex vitamins (B1, B2, B6), provitamin A, that is alpha- and beta-carotene, mixture of other carotenoids (up to 180 mg%), strong antioxidants (tocopherols, tocotrienols), flavonoids (approx. 36 types), fruit acids: malic acid and citric acids, phenolic compounds, approx. 11 mineral salts, including zinc, iron, calcium, selenium, copper, tannins, phospholipids, anthocyanins, steroids, sugars, pectins, approx. 18 amino acids.

**Sea-buckthorn oil has a beneficial effect on skin because:** it is a strong antioxidant – this oil fights free radicals, rebuilds cells and delays cell ageing, supports wound healing, reduces scars and discolourations, treats dermatoses, eczemas, ulceration, psoriasis, atopic dermatitis, acne, improves skin elasticity and structure, provides appropriate hydration of epidermis, limits excessive water loss, protects against harmful radiation (solar or x-rays), has a regenerative and anti-ageing effect.

**Sea-buckthorn oil is significant for human health because:** it supports the function of the immune system, helps to fight infections and microorganisms, improves circulation and heart function, prevents atherosclerosis, lowers the level of cholesterol in blood, supports the function of the digestive system and metabolism, relieves the symptoms of chronic gastric ulcer disease and other diseases of the stomach, duodenum, pancreas, liver and intestines, prevents inflammations, improves the function of brain and the nervous system, lowers the risk of malignant cancers, supports regeneration of the body after chemotherapy and serious diseases, reenergizes and revitalizes, positively affects mood and has an antidepressant effect.

## Conclusions

Sea-buckthorn oil contains an abundance of active substances which is unique in known vegetable oils. Scientific reports confirm the content of almost 200 ingredients which ensure that the oil has a multidirectional effect [3, 7, 8, 12, 14, 15, 67, 82]. Therefore, sea-buckthorn and its oil may be considered to be one of the most valuable natural products in the world. The beneficial effect of various active ingredients contained in sea-buckthorn oil has been recognised in food industry as well as in medicine, pharmacology and cosmetic industry [9, 11, 12, 26, 29, 32, 37, 65] where this oil is used more and more often in skin care preparations or as an adjunctive treatment in various diseases [1, 2, 4, 8–12, 27, 37, 42, 43]. Modern cosmetic and pharmaceutical companies search for natural substances which display unique properties such as sea-buckthorn oil, which added to a product even in a small quantity will undoubtedly ensure its uniqueness.

## Abbreviations

(Z): niem. *Zusammen,* ang. Together
ALA: alpha-linolenic acid
DHA: docosahexaenoic acid
EPA: eicosapentaenoic acid
FA: fatty acids
GLA: gamma-linolenic acid
HDL: high-density lipoprotein
LA: linoleic acid
PUFA: polyunsaturated fatty acids
TEWL: transepidermal water loss
UFA: unsaturated fatty acids
UV: ultraviolet
UVA: ultraviolet A
UVB: ultraviolet B
